# Transition from freshwater to seawater reshapes the skin-associated microbiota of Atlantic salmon

**DOI:** 10.1038/srep19707

**Published:** 2016-01-25

**Authors:** Jep Lokesh, Viswanath Kiron

**Affiliations:** 1 Faculty of Biosciences and Aquaculture, Nord University, 8049 Bodø, Nordland, Norway

## Abstract

Knowledge concerning shifts in microbiota is important in order to elucidate the perturbations in the mucosal barrier during the transitional life stages of the host. In the present study, a 16S rRNA gene sequencing technique was employed to examine the compositional changes and presumptive functions of the skin-associated bacterial communities of Atlantic salmon reared under controlled laboratory conditions and transferred from freshwater to seawater. Proteobacteria was the dominant phylum in salmon from both freshwater (45%) and seawater (above 89%). Bacteroidetes, Actinobacteria, Firmicutes, Cyanobacteria and Verrucomicrobia were the most abundant phyla in salmon from freshwater. The transition to seawater influenced the OTU richness and evenness. The high abundance (~62%) of the genus *Oleispira* made Proteobacteria the most significantly abundant phylum in salmon from seawater. The predictive functional profile suggested that the communities had the ability to extract energy from amino acids in order to maintain their metabolism and scavenge and biosynthesise compounds to make structural changes and carry out signalling for their survival. These findings need to be further explored in relation to metabolic processes, the fish genotype, and the environment.

Mucosal surfaces of animals are colonised by different species of bacteria, archaea, viruses and eukaryotic microorganisms that are either commensals, symbionts or opportunistic pathogens, which are collectively called the microbiome^1^. In aquatic organisms, the mucosal surfaces are the primary protective barriers and harbour both beneficial and opportunistic pathogenic microorganisms^2^. Environmental conditions are known to alter the microbiota of fish^3^. However, the skin-associated microbiota of fish has not been thoroughly investigated, and the interrelationship of the microbiota with the environment is not well known. The shifts in the skin-associated microbiota caused by the environmental changes may lead to the dominance of the opportunistic pathogenic bacteria, which may compromise the health of the fish^4^. This information will be of interest to biologists and the fish farming industry.

Atlantic salmon (*Salmo salar* L.) is one of the major fish species that is grown commercially for human consumption. Salmon are anadromous fish that undergo a physiological process termed smoltification. This process helps juvenile fish living in freshwater adapt to their adult life in seawater (Supplementary Figure S1). In hatcheries, commercial salmon production is performed in two phases: the freshwater phase and the seawater phase. The freshwater phase starts with spawning, followed by egg hatching and rearing until the fish smoltify. Immediately after smoltification, the fish are transferred to seawater. This phase of the transition has been well-studied from physiological, immunological and pathological perspectives^5^. However, knowledge is lacking on the changes in the bacterial communities during this transition.

Skin and its mucosa are the primary barriers in the defence of fish^6^. Any change in the rearing environment of the fish is likely to affect the skin-associated microbial balance^4^,^7^,^8^. Therefore, in the present study we investigated the shift in the skin-associated bacterial community of Atlantic salmon caused by the transfer of the fish from freshwater to seawater. Additionally, we predicted the possible functions of the microbiota of Atlantic salmon maintained under controlled conditions in freshwater and seawater.

## Results

We employed Ion semiconductor sequencing platform to sequence the variable region 4 (Amplicon length 357 bp) of the 16S rRNA gene. This study identified the spatial (freshwater vs seawater) and temporal changes (time points in the seawater phase) in the skin-associated microbiota of Atlantic salmon. A total of 1,613,103 ± 9976 (Mean ± SD) quality-filtered reads were obtained from 38 samples of the four study groups [FW (fish in freshwater), SW.1W (fish 1 week after transfer to seawater), SW.2W (fish 2 weeks after transfer to seawater) and SW.4W (fish 4 weeks after transfer to seawater)], and the data are provided in Supplementary Table S1. The reads obtained from the different groups were classified into 952 OTUs at a 97% similarity level.

### Species richness and diversity of the skin-associated microbiota of Atlantic salmon

**Figure 1:**
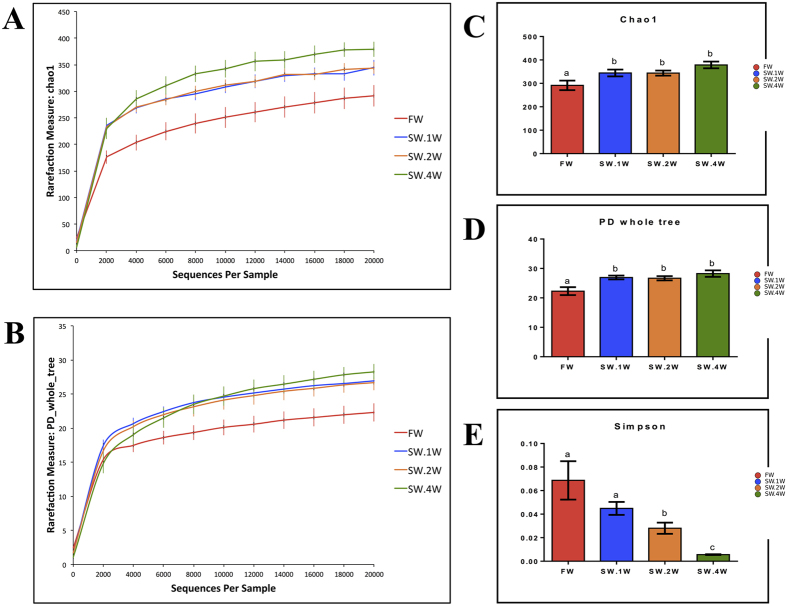
Species richness, phylogenetic diversity and evenness of the skin-associated microbiota of Atlantic salmon. Group-wise averages of the species richness and phylogenetic diversity of the individual samples were employed to prepare the figures. Rarefaction curves on the species richness (Chao1) and diversity (PD whole tree) are shown in A and B, respectively. Significant differences (*p* < *0.05*) in richness, diversity and evenness (Simpson) between groups are indicated using different letters in figures **C**–**E**, respectively. FW - fish in freshwater; SW.1W, SW.2W, SW.4W - fish in seawater, 1, 2 and 4 weeks after transfer. Data are shown as mean ± SEM.

The rarefaction curves based on the alpha diversity metrics [the Chao1 (Fig. 1A) and PD (Phylogenetic diversity) whole tree (Fig. 1B) indices] indicated the adequateness of 20,000 sequences per sample to capture the alpha diversity of the communities. The OTU richness and phylogenetic diversity were significantly higher in SW compared to FW (Fig. 1C,D). Based on the Simpsons evenness index, the OTUs in SW.4W were significantly uneven compared to the communities of the other groups (Fig. 1E). The evenness in SW.1W was similar to that in FW; however, this parameter was significantly lower in the other SW groups (Fig. 1E). These findings are well-supported by the rank abundance analysis (Supplementary Figure S2).

**Figure 2:**
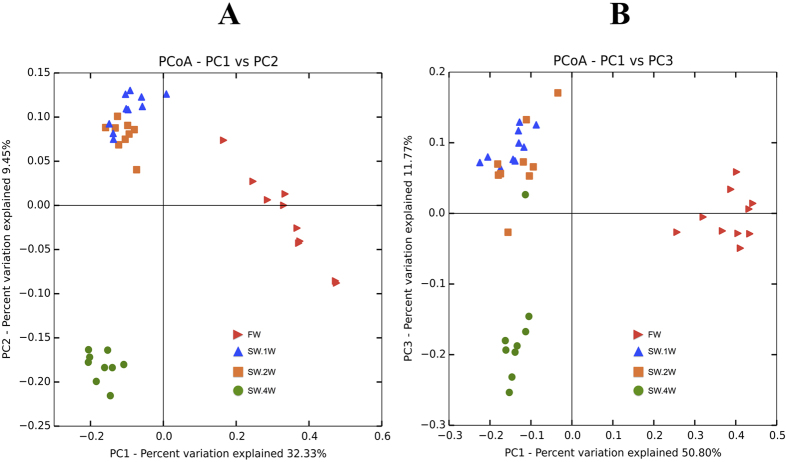
Principle coordinate analysis plot showing differences in the phylogenetic diversities of the skin-associated microbiota of Atlantic salmon. The phylogenetic diversity of the Atlantic salmon skin microbiota was assessed using UniFrac. Unweighted UniFrac distances demonstrate that principle components 1 and 2 capture 41.78% of the variation in FW and SW (**A**). The clustering of the SW.1W and SW.2W samples demonstrates the phylogenetic similarities of their microbiota. The independent clusters of FW and SW.4W are distant from those of SW.1W-SW2W, indicating that they are phylogenetically different from the other groups. A similar grouping pattern is seen in the graph of PC1 vs PC3 (using weighted UniFrac), and the components capture 62.57% of the variation in the microbiota (**B**).

Beta diversity analyses using both unweighted and weighted UniFrac revealed that the FW and SW.4W communities were phylogenetically distant from each other. Furthermore, these communities were phylogenetically distinct from SW.1W and SW.2W. The SW.1W and SW.2W communities clustered together, indicating a phylogenetic similarity between these groups (Fig. 2A,B). UPGMA (Unweighted Pair Group Method with Arithmetic Mean) hierarchical clustering of the weighted UniFrac distances also demonstrated a grouping pattern similar to that obtained by Principal Coordinates Analysis, PCoA (Supplementary Figure S3).

### Relative abundance of the prominent taxa in the skin-associated microbiota of Atlantic salmon

A full list of phyla (abundance ≥ 0.1%) and families (abundance ≥ 0.2%) is provided in Supplementary Tables S2 and S3, respectively. The taxonomic compositions of the skin-associated microbiota of salmon in the freshwater and seawater groups were different based on relative abundances (Supplementary Table S2). In FW, Proteobacteria (45.2%) and Bacteroidetes (39.2%) were among the abundant groups, followed by the Actinobacteria (5.2%), Firmicutes (4.2%) and Cyanobacteria (2.4%). In fish from seawater, Proteobacteria was the dominant phylum (89.2%, 87.4% and 93.5% in SW.1W, SW.2W and SW.4W, respectively), and the abundance of Bacteroidetes, Actinobacteria and the Firmicutes was comparatively lower than their numbers in FW. Flavobacteriaceae (20.3%) was among the abundant families in FW, along with Cytophagaceae (16.4%) and Comamonadaceae (13.6%). The most abundant family in SW.1W was Pseudoalteromonadaceae (14.3%), which increased its dominance by week 2 (29.1%, the dominant type in SW.2W); however, this family had become relatively less dominant by week 4 (5.3%). Campylobacteraceae was the second most abundant group (13.1%) in SW.1W, but its representation dropped by week 2 (1.2%) and increased again to 8.1% by week 4. In SW.2W, Oceanospirillaceae was the second most abundant type (9.2% abundance in SW.1W); it became the dominant family in SW.4W by week 4 due to an increase in its percentage representation from 15.4% to 62.8%. In SW.1W and SW.2W, the third, fourth and fifth most abundant groups belonged to Gammaproteobacteria. In SW.1W, Colwelliaceae (10.7%) was the fourth most dominant type; its representation decreased to 6.7% by week 2 and increased again to 8.7% to become the second most dominant type in SW.4W.

### Differential abundances of the OTUs in the skin-associated microbiota of Atlantic salmon

**Figure 3:**
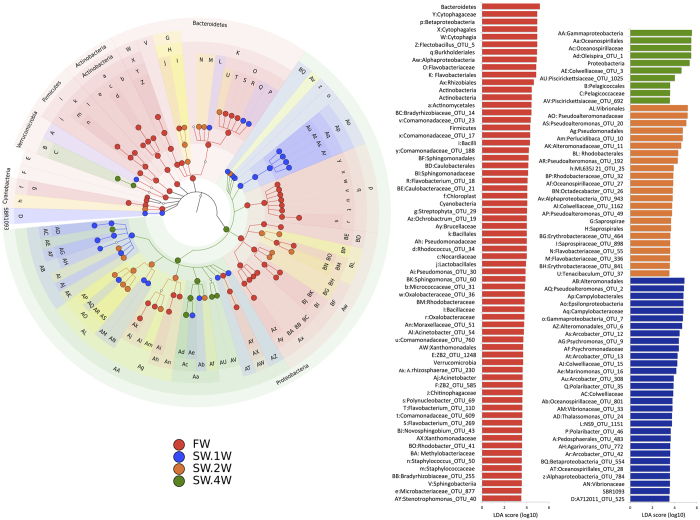
Cladogram showing differentially abundant taxa of the skin-associated microbiota of Atlantic salmon. LEfSe was used to validate the statistical significance and the effect size of the differential abundances of taxa in FW, SW.1W, SW.2W and SW.4W. The 4 different groups were treated as 4 classes and a multiclass comparison was performed with the Kruskal-Wallis and Wilcoxon rank-sum tests, with a p-value cut-off of 0.05 and an LDA score cut-off of 3.5. Differentially abundant taxa are colour-coded by class (different study groups). In the cladogram, OTUs associated with the bacteria from different study groups are represented using letters. The taxonomic level identities corresponding to the letters are given alongside the LDA score bar. Taxonomic names given in the figure are based on the Greengenes database (release gg_13_8).

LEfSe [Linear Discriminant Analysis (LDA) Effect Size] was used to detect the bacterial taxonomic biomarkers in FW and SW, resulting in the identification of 129 clades including both lowly and highly abundant bacteria. The significantly abundant taxa are shown in Fig. 3, with their corresponding LDA scores. Proteobacteria, Bacteriodetes, Actinobacteria, Firmicutes, Verrucomicrobia, Cyanobacteria, and SBR1093 were the phylum-level biomarkers. Of these, all except Proteobacteria and SBR1093 were biomarkers of FW. Family-, genus- and species-level biomarkers are provided in the Supplementary Table S4.

The class-level biomarkers of Proteobacteria in FW belonged to Alpha- and Betaproteobacteria. In contrast, Gammaproteobacteria was the most abundant class in SW.4W; its dominance was driven by the highly abundant *Oleispira* OTU1. Epsilonproteobacteria was the most significantly abundant class in SW.1W. Additionally, one OTU each belonging to the Alpha-, Beta- and Gammaproteobacteria was abundant in SW.1W. At the class-level, only 1 OTU (belonging to Alphaproteobacteria) was significantly abundant in SW.2W.

Thirteen order-level biomarkers under the phylum Proteobacteria were identified by LEfSe. These included five features in FW (Burkholderiales, Caulobacterales, Sphingomonadales, Rhizobiales, and Xanthomonadales), 4 in SW.1W (Campylobacteriales, Alteromonadales and one OTU each from Oceanospirillales and Alteromonadales), 3 in SW.2W (Vibrionales, Pseudomonadales, and Rhodobacterales) and 1 in SW.4W (Oceanospirillales).

The phylum-level abundance of Bacteroidetes in FW was driven by class-level (Sphingobacteriia and Cytophagia) and order-level (Flavobacteriales and Cytophagales) biomarkers. All of the biomarkers identified under Actinobacteria and Firmicutes belonged to the FW group. The class-level biomarkers under the phyla Actinobacteria and Firmicutes were identified as Actinobacteria and Bacilli, respectively. At the order level, only the Actinomycetales biomarker was under Actinobacteria. In contrast, the phylum Firmicutes had 2 biomarkers (Lactobacillales and Bacillales). Although the phylum-level abundance of Verrucomicrobia was evident in FW, the order-level biomarkers in Verrucomicrobia present in SW.1W (Pedosphaerales) and SW.4W (Pelagicoccales) did not belong to FW.

### Core and shared OTUs in the different fish groups

**Figure 4:**
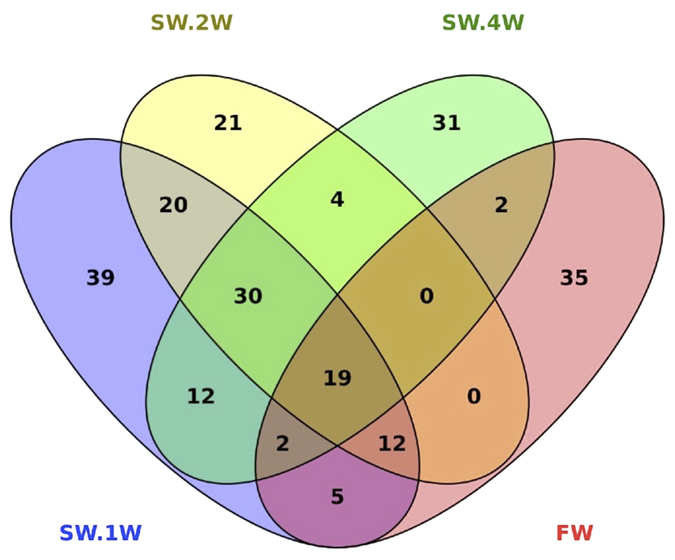
Shared and unique core OTUs in the skin-associated microbiota of Atlantic salmon. To evaluate the number of unique and shared OTUs in FW and SW, a core microbiota (OTUs that were present in at least 90% of the samples in each group) was computed. VENNY 2.0.2^63^ was used to identify the unique and shared OTUs in the different study groups. A Venn diagram shows the numbers of shared and unique core OTUs in the microbiota of the FW and SW groups.

The shared and unique core OTUs shown in Fig. 4 are based on the OTUs present in 90% of the samples of a particular group (i.e. 9 fish out of 9 or 10). Interestingly, 19 core OTUs were common in all 4 groups (Supplementary Table S5). Although these OTUs were present in most samples, it is important to note that there was a significant difference in their abundance across groups.

### Presumptive metabolic potential of the skin-associated microbiota of Atlantic salmon

**Figure 5:**
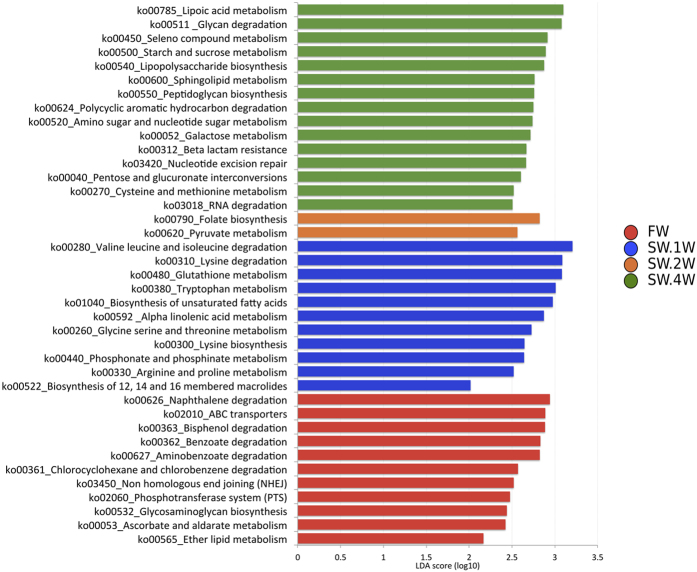
Differentially abundant presumptive functions of the skin-associated microbiota of Atlantic salmon. PICRUSt was used to predict the metagenome, and HUMAnN was employed to find the associated metabolic pathways. The significantly abundant pathways were identified with the help of LEfSe (p-value cut-off of 0.05 and LDA score cut-off of 2).

Eleven functional pathways were enriched in both FW and SW.1W. Two pathways were abundant in SW.2W, and 15 significantly enriched pathways were found in SW.4W. The results are graphically represented using LDA scores in Fig. 5.

## Discussion

In Atlantic salmon hatcheries, fry and parr are reared in freshwater. After smoltification, they are released into seawater. A change in the rearing environment is likely to alter the bacterial community on the skin of farmed salmon. This hypothesis was tested in the present study. The richness, diversity and taxonomic composition of the skin-associated microbiota of farmed Atlantic salmon were evaluated in freshwater and seawater. Additionally, the taxa shift during the 4-week seawater transition period and significant differences in the predicted metabolic potential of the microbial community in the two rearing environments were assessed.

In the present study, the approach was to compare the microbiota of the fish from the two treatments - the differential abundance and diversity of the bacterial taxa on the fish mucus is discussed. However, the presence and importance of treatment-specific bacteria on salmon mucus have to be interpreted with caution since water samples were not analysed and there were no tank replicates (i.e., for the treatments).

The higher evenness in FW could indicate that a stable bacterial community was established during the host’s life in freshwater (8 months in the hatchery and 2 months in the research station) because higher evenness is a feature of the functional stability and resilience of the community^9^. The evenness decreased after entry into seawater, and higher richness and phylogenetic diversity became the characteristics of the seawater community. This transition is most likely a protracted process, possibly taking longer than the 4-week time line examined in the present study. However, the presence of few dominant OTUs is the characteristic feature of the marine fish microbiota^10^. In terms of the phylogenetic similarities/differences, the FW community was distinct from its seawater counterparts, and the SW.4W community was different from the SW.1W and SW.2W communities. Taken together, these results suggest that the transfer of the fish from freshwater to seawater may disturb the skin-associated community of Atlantic salmon.

The phylum Proteobacteria had a high mean percentage representation in all of the study groups. However, the representation of the communities belonging to Proteobacteria increased significantly upon entry into seawater. The second largest representative phylum in FW was Bacteriodetes (39.2%), which decreased significantly in fish reared in seawater. These results and the high representation of the members of Proteobacteria in the shared core microbiota could indicate the importance of this phylum in the skin-associated communities of Atlantic salmon. Psychrophiles are mostly Gram-negative Proteobacteria^11^, and therefore, it is not surprising to find Proteobacteria as the dominant phylum in a cold water fish. In fact, none of the other significantly abundant phyla in FW (except Cyanobacteria) were present among the shared core OTUs. Furthermore, the significant abundance of the members of the phylum Proteobacteria (class- to species-level) was not translated to the phylum-level abundance. The high abundance (~62%) of the genus *Oleispira* in SW.4W (Fig. 3) made Proteobacteria the most significantly abundant phylum in the seawater group. *Oleispira* was also a shared core OTU (Supplementary Table S5). A species of *Oleispira* (*O. antarctica*) was described as a psychrophilic hydrocarbonoclastic bacteria^12^. The high abundance of bacteria belonging to this genus in salmon skin is intriguing, and their presence in both the freshwater and seawater phases could indicate their facultative nature.

The class- to species-level (9 genera in FW and SW.1W and 5 genera in SW.2W) differential abundance of Proteobacteria was noted in FW, SW.1W and SW.2W. A phylum-level differential abundance of Proteobacteria was detected in SW.4W. Moreover, although SW.1W and FW had a similar number of generic shifts, 4 OTUs of the same genus (*Arcobacter*) were significantly more abundant in SW.1W. Similarly, 3 OTUs of the same genus (*Pseudoalteromonas*) were differentially abundant in SW.2W (Fig. 3). These results may imply that these genera are the major drivers of the differences at this level of the taxon. Furthermore, *Arcobacter* and *Pseudoalteromonas* were present in the shared core OTUs (Supplementary Table S5). Some species of the genus *Pseudoalteromonas* are capable of synthesising extracellular compounds that can inhibit the colonisation of competing microbes^13^. Therefore, these microbes might have outcompeted other communities due to their ability to produce antimicrobial compounds. Conversely, species belonging to the genus *Arcobacter* are reported to be harmful to the host. The pathological effects of *Arcobacter cryaerophilus* on rainbow trout (*Oncorhynchus mykis*s) were reported previously^14^. Among several species of *Pseudoalteromonas* associated with cultured gilthead sea bream (*Sparus aurata*) and European sea bass (*Dicentrarchus labrax*), only one strain was pathogenic for the gilthead sea bream and one strain was weakly virulent for the sea bass^15^. The significant abundance of these two types of skin-associated bacteria in Atlantic salmon during the transition is interesting, particularly from a disease point of view. The characteristics of species belonging to *Stenotrophomonas* (FW) and *Psychromonas* (SW.1W), which were the two other core OTUs and genus-level biomarkers, were described by others: *Psychromonas arctica* is a biofilm-forming cold-tolerant bacterium isolated from seawater and sea ice^11^, and; *Stenotrophomonas maltophilia* is associated with infectious intussusception syndrome in the channel catfish *Ictalurus punctatus*^16^.

Other genera under Proteobacteria (i.e., *Polynucleobacter*, *Rhodobacter*, *Sphingomonas*, *Novosphingobium*, *Ochrobactrum*, *Pseudomonas*, and *Acinetobacter*) were also significantly abundant in FW. Association of the members of these genera with either aquatic environments or aquatic organisms has been reported previously. *Polynucleobacter necessarius asymbioticus* is known for its ubiquitous presence in lentic freshwater habitats^17^. *Rhodobacter veldkampii* was isolated from the gill samples of Atlantic salmon subjected to a freshwater bath to prevent gill disease caused by *Neoparamoeba pemaquidensis*^18^. The genera, *Sphingomonas* and *Novosphingobium*, were found in the intestine of farmed rainbow trout^19^, and they assisted in the metabolism of nitrogenous compounds^20^. Furthermore, they include species that can degrade aromatic compounds^21^,^22^. In the present study, pathways related to the degradation of aromatic compounds were found to be enriched in FW, and these 2 genera might have been the key contributors. *Ochrobactrum* was found to be associated with both polluted soil^23^ and the intestine of Atlantic cod^24^. The *Pseudomonas* genus was present in both the skin and gills of Atlantic salmon^18^. Two commensal bacteria belonging to *Pseudomonas* and *Psychrobacter* were isolated from wild Atlantic cod^25^. *Acinetobacter* was also present in the skin and gills of Atlantic salmon^18^. The species *Acinetobacter rhizosphaerae* is another biomarker of the FW group.

*Thalassomonas*, *Psychromonas*, *Agarivorans*, *Pseudoalteromonas*, *Marinomonas*, *Arcobacter*, *Perlucidibaca*, *Octadecabacter*, and *Oleispira* (all belonging to the phylum Proteobacteria) were the biomarkers of the SW groups. Previous reports indicated the presence of members belonging to the aforementioned genera in the intestine of wild-caught shrimp (*Thalassomonas* sp.)^26^, Atlantic halibut larvae (*Marinomonas* and *Pseudomonas*)^27^, sediments of aquaculture sites (*Agarivorans albus*)^28^, freshwater (*Perlucidibaca piscinae*)^29^, and Antarctic sea and Arctic sea ice (*Octadecabacter*)^30^.

Bacteroidetes was the second dominant type in all groups, and a phylum-level abundance was noted in FW. Furthermore, Firmicutes, Actinobacteria, Verrucomicrobia and Cyanobacteria were significantly abundant in FW. Several genera (i.e., *Flectobacillus*, *Flavobacterium*, *Polaribacter*, *Tenacibaculum*, *Rhodococcus*, and *Staphylococcus*) contributed to these phylum-level abundances in the FW and SW groups. The functional roles of the members of these bacterial genera that are present in the mucus of Atlantic salmon have to be ascertained through further studies, especially because some of them are known fish pathogens^31^,^32^,^33^,^34^. On the other hand, some of the bacteria could be part of a healthy skin microbiome of the fish.

The genus-level biomarkers indicate that the FW group is characterised by the significant abundance of several members of Proteobacteria, Bacteriodetes, Actinobacteria and Firmicutes, and some species under these phyla also include known opportunistic bacteria. Although diverse bacteria colonised the skin of the SW groups, only a few clades were found to be significantly abundant compared to the FW group. Atlantic salmon had a significant abundance of clades that include opportunistic bacteria belonging to Proteobacteria and Bacteriodetes in their skin, even 1 week after transfer to seawater. However, the composition of such opportunistic clades decreased by the second week. Furthermore, the significant abundance of the genus of a psychrophilic Proteobacteria in SW.4W could be indicative of the ability of a cold-tolerant bacterial type to establish its dominance in its niche.

The global changes in the presumptive functions of the skin-associated microbial community of Atlantic salmon were also examined by predicting the metagenomes using PICRUSt. The accuracy of the prediction was evaluated by computing NSTI (Nearest Sequenced Taxon Index) - the NSTI scores ranged between 0.10 and 0.17. Eleven functional pathways were found to be abundant in FW, including those related to the metabolism of ether lipid and ascorbate-aldarate, biosynthesis of glycosaminoglycan, phosphotransferase system (PTS), non-homologous end joining (NHEJ), ATP-binding cassette (ABC) transporter, and degradation of several aromatic compounds.

The skin microbiota of Atlantic salmon in week 1 of the seawater phase was linked to 11 pathways: metabolism of arginine, proline, glutathione, glycine, serine, threonine, tryptophan, phosphonate, phosphinate, α-linolenic acid; biosynthesis of lysine, 12-, 14-, 16-membered macrolides and unsaturated fatty acids; and degradation of lysine and branched-chain amino acids (valine, leucine and isoleucine). Bacteria can metabolise amino acids or fatty acids to derive energy for their growth^35^. Some bacteria such as lactic acid bacteria (LAB) can derive pyruvate and lactate from carbohydrates, organic acids and amino acids, with amino acids serving as the main substrates in this process. The pathways may indicate the ability of the bacteria to extract energy from amino acids and maintain their metabolism during adaptation to seawater. Glutathione reserves are used by the bacteria to protect their cells from external pressures, such as low pH, cold, and oxidative and osmotic stresses^36^.

Fifteen pathways were attributed to the skin microbiota of Atlantic salmon in seawater after 4 weeks. This finding indicates that the SW.4W group has bacteria that can synthesise and/or scavenge lipoate^37^ and metabolise selenite into selenomethionine, selenocysteine/selenocystine, and selenocystathionine^38^. The bacterial community can use sulfate/thiosulfate/sulfonates/methionine/cysteine-derived compounds (glutathione) as sulfur sources, use serine to synthesise cysteine, or take up cysteine directly from the environment via ABC transporters or symporters^39^. The microorganisms can biosynthesise and transport methionine^40^,^41^ and synthesise physical barrier-providing molecules called peptidoglycans^42^. Furthermore, they are able to metabolise amino sugars and nucleotide sugars, possibly for cell wall/exopolysaccharide synthesis^43^. The members of the community can form biofilms via galactose metabolism^44^, produce lipopolysaccharides (LPS) to maintain the outer-membrane permeability barrier integrity and contribute to host-pathogen interactions by effectively synthesising and exporting LPS^45^,^46^. The identified members can also metabolise sphingolipids (membrane constituents and cell signalling molecules) and survive using sphingolipid signalling^47^,^48^. Thus, the skin-associated bacteria of Atlantic salmon reared in seawater could possibly scavenge and biosynthesise compounds to make structural changes and carry out signalling for their survival.

## Conclusion

The dominance of Proteobacteria in the skin-associated microbiota of Atlantic salmon is evident from the current findings. The transition from freshwater to seawater destabilised the skin bacterial community, leading to an increase in phylogenetic diversity in the fish mucus of Atlantic salmon in seawater. The significant abundance of the Proteobacteria phylum in freshwater fish was driven by multiple clades (downwards from the class-level). The phyla Bacteriodetes, Actinobacteria, Firmicutes, Verrucomicrobia and Cyanobacteria were also significantly abundant in the freshwater fish. Although diverse bacteria colonise the skin of Atlantic salmon in seawater, only a few clades (Proteobacteria, Bacteriodetes, Cyanobacteria, Verrucomicrobia and SBR1093) were found to be significantly abundant. The abundance of some clades, which include known opportunistic bacteria, in the freshwater fish appeared to diminish during the switch to seawater life. The roles of these bacteria can be explained only after conducting functional studies. The dominance of the psychrophilic genus *Oleispira* in fish adapted to seawater needs to be ascertained in order to clarify the importance of the bacteria. The presumptive functional content of the bacterial community during the transition stages could indicate their ability to extract energy from amino acids to maintain their metabolism and scavenge and biosynthesise compounds to make structural changes and carry out signalling for their survival. The community profiles revealed in this study could facilitate further investigations on specific bacterial groups to reveal their relevance to Atlantic salmon during the physiological changes accompanying smoltification. The significant findings need to be further explored in relation to metabolic processes, the fish genotype, and the environment.

## Methods

### Study design

Atlantic salmon (0 year class from the freshwater production stage), obtained from the hatchery of Cermaq Norway AS (Bodø, Norway), were maintained at the Research Station, Nord University, Norway. They were reared in 500 L tanks that were part of a freshwater (city water supply, 12 °C) flow-through system, and were offered a commercial feed from EWOS (Bergen, Norway). The fish were maintained in freshwater, at the Research Station, for approximately 2 months before the sampling. Initial samples (FW) were collected from these fish, and then fifty fish were transferred to the seawater (drawn from a depth of 50 m in Saltfjorden) tank (500 L) of another flow-through system (12 °C). Following introduction of the fish into seawater (salinity 35 g/kg), samples were obtained at 1 (SW.1W), 2 (SW.2W) and 4 weeks (SW.4W). During this period, the fish were fed the same diet mentioned above. The experiment was conducted under controlled conditions: the photoperiod in the laboratory was 12L:12D, the temperature of the rearing water was 12 °C, and the dissolved oxygen was above 90%. The water used in both the FW and SW systems was initially filtered through a drum filter (25 micron), and then passed through a protein skimmer. Next, the water was treated with UV light and finally filtered through a 6 micron filter.

### Sampling

The fish were anaesthetised using MS-222 (80 mg/L Tricaine methanesulphonate, Argent Chemical Laboratories, Redmond, WA, USA) prior to sampling. At each sampling time point, skin mucus samples (n = 9 or 10 fish; mean ± SD = 88.8 ± 2.8 g) were gently scraped from the entire body surface of the fish (excluding the regions close to the anus and the gill opening) using sterile glass slides. The mucus samples were transferred to cryotubes, flash frozen in liquid nitrogen, and stored at –80 °C prior to processing. The handling and sampling procedures were conducted according to the authorised protocols of the Norwegian Animal Research Authority (FDU), and the study was approved by FDU (approval number: 7899).

### DNA extraction

DNA from the samples was extracted using the QIAamp DNA Stool Mini Kit (Qiagen, Nydalen, Sweden). The samples were mixed with ASL buffer from the kit at a 1:10 ratio and vortexed using the Vortex Gene 2 (Scientific Industries, Bohemia, NY, USA). The manufacturer’s protocol was slightly modified to improve the lysis of cells and increase the DNA yield. The modifications were as follows: in step 1, the samples were homogenised with the ASL buffer for 10 min, and in step 3, the samples were heated at 70 °C for 10 min.

### PCR amplification of the V4 region of the 16S rRNA gene and sequencing

The DNA concentration of each sample was measured using the Qubit® dsDNA BR Assay Kit (Life Technologies, Grand Island, NY, USA) in combination with the Qubit® 2.0 Fluorometer (Life Technologies). The V4 region of the 16S rRNA gene was amplified by the fusion primer method using the primers 515F (GTGCCAGCMGCCGCGGTAA) and 806R (GGACTACHVGGGTWTCTAAT). These primers were shown to be ideal to amplify the V4 region with high coverage, and the amplicons (read length) are suitable for the Ion Torrent™ sequencing platform (Life Technologies)^49^. Variable region 4 was selected because sequencing and taxonomic assignment using this region was associated with a low error rate and minimum loss of taxonomic resolution^50^,^51^. The gene- specific primers were modified in the following manner: forward primer = 5′ Ion adapter A (CCATCTCATCCCTGCGTGTCTCCGAC)/4-base key (TCAG)/Ion Xpress™ barcode (sample specific)/3-base separator (GAT)/515F (GTGCCAGCMGCCGCGGTAA) 3′ and reverse primer = 5′ Ion adapter trp1 (CCTCTCTATGGGCAGTCGGTGAT)/806R (GGACTACHVGGGTWTCTAAT) 3′. Full-length primer sequences (including the barcodes) that were used for the amplification of the V4 region in the individual samples are listed in Supplementary Table S1. All PCR reactions were performed in duplicate in a 50 μl reaction mixture, comprising 45 μl of Platinum® PCR SuperMix High Fidelity (Life Technologies), 1 μl of the sample-specific primer mix (200 nM), and 4 μl of the DNA template (~200 ng). A negative PCR control without the DNA template was also included in the run. Thermocycling conditions included an initial denaturation at 94 °C for 5 min, followed by 35 cycles of denaturation at 94 °C for 30 sec, annealing at 56 °C for 30 sec and extension at 68 °C for 45 sec. After the cycling procedure, the PCR products corresponding to each sample were pooled and run on a 1.2% agarose gel for the separation of amplicons from the leftover primers and primer-dimers. The positive bands (~357 bp) were excised from the gel and purified using the E.Z.N.A. Gel Extraction Kit (Omega bio-tek, Norcross, GA, USA). The negative control did not show any amplification.

Amplicon libraries were quantified using the KAPA Library Quantification Kit (Kapa Biosystems, Woburn, MA, USA) for the Ion Torrent™ platform according to the manufacturer’s protocol. Briefly, each amplicon library was serially diluted (1:500, 1:1000, 1:2000 and 1:4000), and qPCR was performed on each of the dilutions of the libraries in a reaction mixture comprising the KAPA SYBR FAST qPCR master mix containing the primer premix (12 μl), PCR-grade water (4 μl) and the diluted library or DNA standard (4 μl). The Cq values corresponding to the different libraries and the values corresponding to the DNA standards were used to calculate the size-corrected dilution factor for each sample as explained in the kit protocol. Each amplicon library was subsequently diluted with low TE buffer to obtain a concentration of 26 pM (~15.6 million molecules/μl). Then, 5 μl of each of the diluted libraries (26 pM) was pooled prior to the emulsion PCR. The emulsion PCR was performed using the Ion PGM™ Template OT2 400 Kit (Life Technologies) following the manufacturer’s instructions. The libraries were sequenced using the Ion 318™ Chip Kit v2 (Life Technologies) on the Ion Torrent PGM™ system employing the Ion PGM™ Sequencing 400 Kit (Life Technologies). Initial quality filtration of the sequence data, such as the removal of polyclonal and low quality reads, adapter sequences and the barcode sequences, was performed by the Torrent Suite™ Software (Life Technologies). The FASTQ files corresponding to each sample were used for analysis.

### Data analyses

The 16S rRNA gene data were analysed using QIIME version 1.8.0^52^ and UPARSE version 7.0.1090^53^. Sequences were first quality filtered by discarding reads: i) that fell outside the length range of 290 to 300 bp; ii) with a Phred score below 25 over a window of 50 nucleotides; and iii) with homopolymers and ambiguous base-pairs exceeding 6. The resulting quality-filtered FASTA files were merged. Then, the sequences were trimmed to 250 bp, dereplicated, and abundance sorted, and sequences with <10 reads were discarded. A *de novo* OTU-picking was performed on the quality-filtered and sorted sequences. Subsequently, UCHIME version 4.2.40 was employed to perform a reference-based chimaera check^54^. The reads were mapped to the OTUs by searching the reads as a query set against the OTU representative sequences. Only OTUs present in at least 3 samples with an abundance ≥0.001% were retained for further analysis. The sequences were assigned to the lowest possible taxonomic rank using UCLUST version 1.2.22^55^ by considering ten database hits with a similarity level of 0.9 employing the Greengenes^56^ reference database (release gg_13_8). A phylogenetic tree was constructed using FastTree version 2.1.7^57^. Then, alpha diversity metrics (PD whole tree, Chao1 index and Simpson’s evenness index) were computed. Statistical analyses of the alpha diversity indices were performed using GraphPad Prism 6 (GraphPad Software, Inc., La Jolla, CA, USA). The assumptions of the t-test and ANOVA were checked before performing the analyses. An unpaired t-test (with Welch’s correction when necessary) was used to find the spatial effects (FW vs each of the SW stages). To elucidate the temporal effects of the SW stages, one-way ANOVA followed by Tukey’s multiple comparison test was employed. Statistical significance was established at *p < 0.05*. Beta diversity was measured using UniFrac^58^, and PCoA was performed on the UniFrac results.

Differential abundance of the OTUs across different groups was assessed using LEfSe version 1.0^59^ to elucidate both the spatial and temporal differences, with the number of sequences rarefied to 20,000 per sample, the *p-value* set at *0.05* and an LDA log score threshold of 3.5. The results were plotted in the form of a cladogram using GraPhlAn version 0.9.7^60^. To predict the metagenomes of each of the samples, a closed reference (gg_13_5) OTU-picking strategy was adopted with a 97% sequence similarity threshold using PICRUSt version 1.0.0 with the default parameters^61^. The accuracy of the predictions of the metagenomes was assessed by computing NSTI (Nearest Sequenced Taxon Index), which is an index that indicates the relation of the microbes in a particular sample to the bacterial genomes in a database. The NSTI score corresponding to the PICRUSt (Phylogenetic Investigation of Communities by Reconstruction of Unobserved States) analysis is provided in Supplementary Table S6. The associated metabolic pathways were deciphered by employing HUMAnN (The HMP Unified Metabolic Analysis Network) version 0.99 with the default settings^62^. The identified pathways were analysed for statistical significance using LEfSe with a p-value cut-off of 0.05 and an LDA log score cut-off of 2.

Core OTUs that were present in at least 90% of the samples in each group (9 fish per group) were also computed using QIIME. The number of core OTUs shared between groups was identified using VENNY version 2.0.2^63^. The raw data could be accessed under the MG-RAST IDs: 4626776.3, 4626778.3, 4626779.3, 4626780.3, 4626781.3, 4626782.3, 4626783.3, 4626784.3, 4626785.3, 4626777.3, 4626786.3, 4626788.3, 4626789.3, 4626790.3, 4626791.3, 4626792.3, 4626793.3, 4626794.3, 4626795.3, 4626787.3, 4626796.3, 4626797.3, 4626798.3, 4626799.3, 4626800.3, 4626801.3, 4626802.3, 4626803.3, 4626804.3, 4626805.3, 4626806.3, 4626807.3, 4626808.3, 4626809.3, 4626810.3, 4626811.3, 4626812.3 and 4626813.3.

## Additional Information

**How to cite this article**: Lokesh, J. and Kiron, V. Transition from freshwater to seawater reshapes the skin-associated microbiota of Atlantic salmon. *Sci. Rep*. **6**, 19707; doi: 10.1038/srep19707 (2016).

## Supplementary Material

Supplementary Information

Supplementary Tables
