# Supplementary material for: Transition from freshwater to seawater reshapes the skin-associated microbiota of Atlantic salmon
Source: Sci Rep. 2016 Jan 25;6:19707. doi: 10.1038/srep19707 (PMC4726331; doi:10.1038/srep19707)
Supplement: Supplementary Information [file srep19707-s1.pdf]

Supplementary figures

Transition from freshwater to seawater reshapes the skin-associated microbiota of Atlantic salmon

Jep Lokesh, Viswanath Kiron

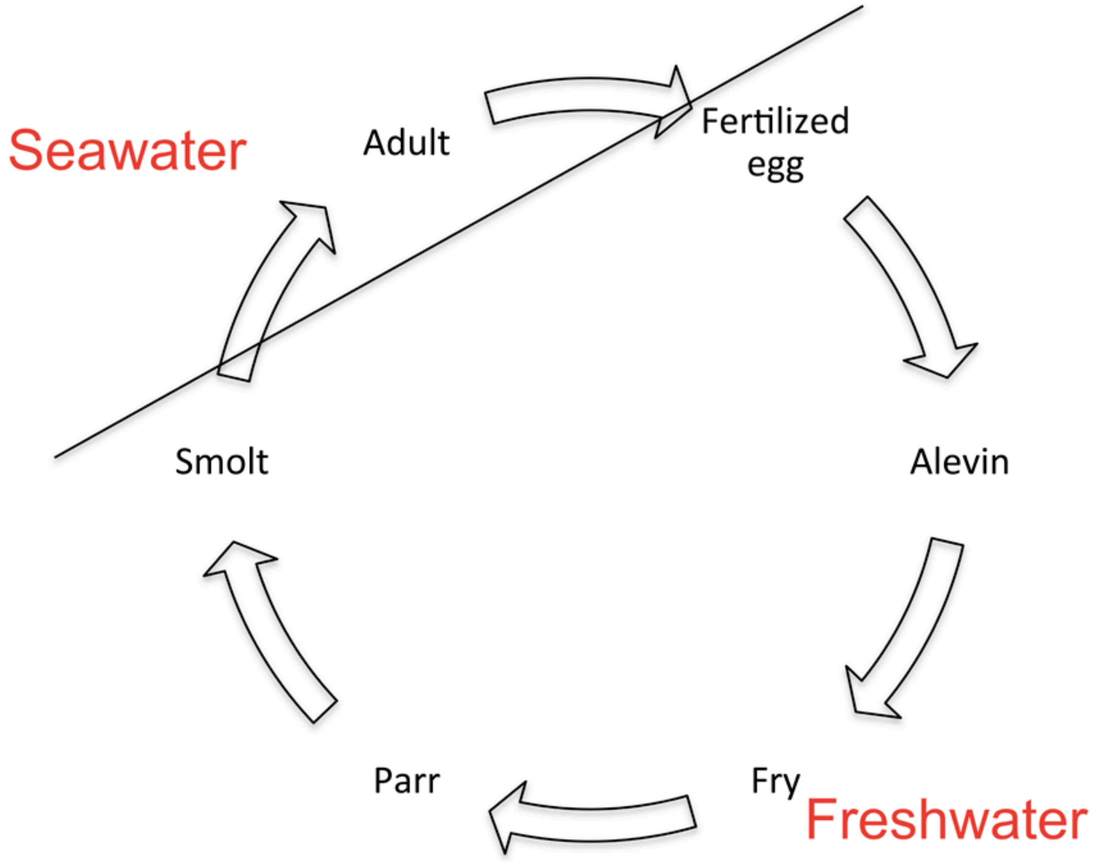

Supplementary Figure S1: Life cycle of Atlantic salmon under aquaculture.

Fertilized eggs of Atlantic salmon are hatched and grown until the fish reach smolt stage (~50-100g). Once the smoltification is completed, they are transferred to the seawater where they are grown to market size (~4-5Kg).

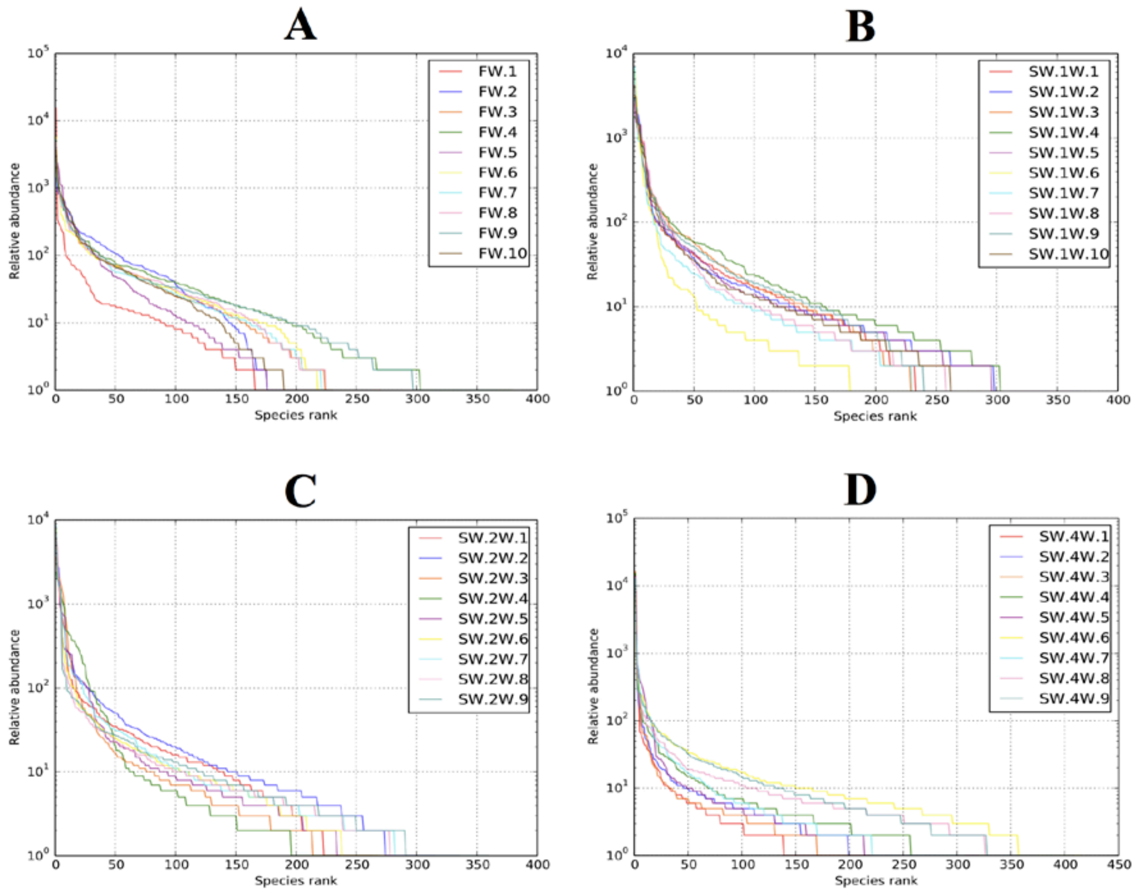

Supplementary Figure S2: Rank abundance plot. Plot showing the ranking of abundances of different OTUs in FW (A), SW.1W (B), SW.2W (C) and SW.4W (D) groups.

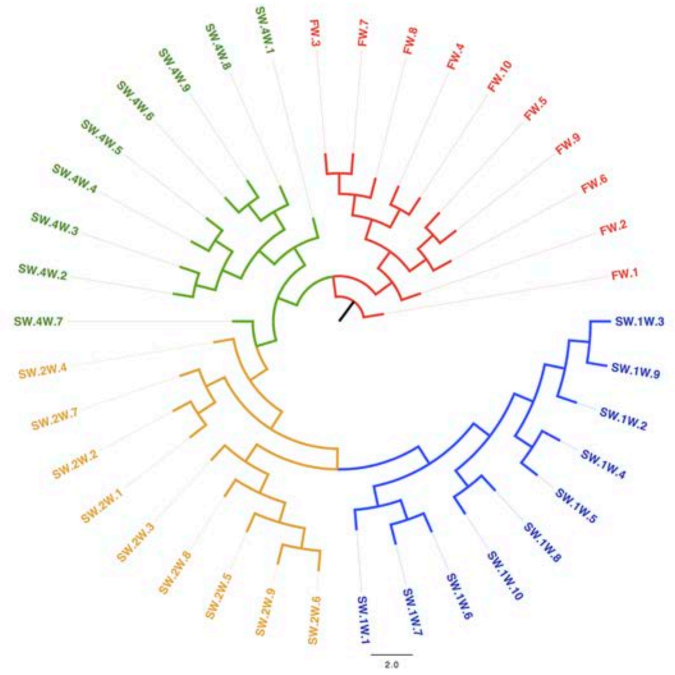

Supplementary Figure S3: Hierarchical clustering of Atlantic salmon skin microbiota. UPGMA (Unweighted Pair Group Method with Arithmetic Mean) hierarchical clustering on the weighted UniFrac distances also demonstrated a trend similar to that obtained by PCoA.
